# Comparison of metabolic and functional parameters using cardiac 18F-FDG-PET in early to mid-adulthood male and female mice

**DOI:** 10.1186/s13550-021-00748-z

**Published:** 2021-01-19

**Authors:** Maximilian Fischer, Mathias J. Zacherl, Tobias Weinberger, Ludwig Weckbach, Bruno Huber, Christian Schulz, Steffen Massberg, Peter Bartenstein, Sebastian Lehner, Andrei Todica

**Affiliations:** 1 Medizinische Klinik und Poliklinik I, Klinikum der Universität München, Ludwig-Maximilians-Universität, Marchioninistrasse 15, 81377 Munich, Germany; 2 Department of Nuclear Medicine, University Hospital, LMU Munich, Marchioninistr. 15, 81377 Munich, Germany; 3 Ambulatory Healthcare Center Dr. Neumaier & Colleagues, Radiology, Nuclear Medicine, Radiation Therapy, Regensburg, Germany

**Keywords:** Cardiac positron emission tomography, 18F-FDG, Heart function, Heart physiology

## Abstract

**Background:**

In this descriptive study of male and female mice at different weeks of age, we use serial non-invasive cardiac 18F-FDG-PET scans to follow up on metabolic alterations, heart function parameters, and the ECG of both sexes in early to mid-adulthood.

**Methods:**

ECG-gated 18F-FDG-PET scans were performed in mice on 10, 14, and 18 weeks of age, using a dedicated small-animal PET scanner. The percentage of the injected activity per gram (%IA/g) in the heart, left ventricular metabolic volume (LVMV), myocardial viability and left ventricular function parameters: end-diastolic (EDV), end-systolic (ESV), stroke volume (SV), and the ejection fraction (EF%) were estimated.

**Results:**

Compared to their age-matched female counterpart, male mice showed a constant increase in LVMV and ventricular volume during the follow-up. In contrast, female mice remain stable after ten weeks of age. Furthermore, male mice showed lower heart rates, positive correlation with cardiac %IA/g, and negative correlation with LVMV.

**Conclusion:**

In this study of serial cardiac PET scans, we provide insight for basic murine research models, showing that mice gender and age show distinct cardiac metabolisms. These physiologic alterations need to be considered when planning in vivo injury models to avoid potential pitfalls.

## Introduction

Cardiomyopathy, heart failure, and coronary artery disease are the main reasons for heart diseases being the leading causes of death in western society [1]. Fundamental in vitro and in vivo molecular research aims to find new translational diagnostics and therapeutic perspectives to lower mortality and improve patients' quality of life. Especially in vivo animal models are most suitable for transferring clinical obstacles, such as myocardial infarction due to coronary artery disease into a research setting to decipher underlying mechanisms and prevent further deteriorating events. For instance, murine models of myocardial infarction aim to reduce the myocardial damage and improve the cardiac remodelling to preserve the cardiac function after irreversible loss of heart muscle cells.

Standard animal models, including mice, rats, rabbits, and pigs, are used to broaden our understanding of diseases. Out of these, mice models are the most common due to short reproduction time, comfortable breeding, feasible logistics, and costs.

Most of the current cardiac injury models are performed with male mice [2, 3]. Nevertheless, several murine studies indicate that cardiovascular injury models, depending on mouse gender, differ in their results [4, 5].

For instance, mice deficient of the G-protein coupled receptor 30 (GPR30), a membrane-bound estrogen receptor involved in the estradiol signalling, demonstrate that male but not female mice develop impaired left-ventricular cardiac function [6]. The overexpression of the cardiac sodium-calcium exchanger increases the susceptibility to ischemia–reperfusion injury in male but not female mice [7]. In a diabetic mouse model, the induced adverse cardiac remodelling was observed earlier in female mice than in their male counterparts [8]. These publications further state several underlying molecular and functional differences in the hearts of male and female mice.

Moreover, mice are used for cardiac injury models at different age stages, commonly starting at eight weeks up to 18 weeks.

Functional imaging using positron emission tomography (PET) facilitates serial non-invasive measurements of the same individuum at different time points [9, 10]. ECG-gated functional PET imaging with 2-deoxy-2-[^18^F]fluoro-D-glucose (18F-FDG) not only enables the assessment of cardiac viability but also estimating left ventricular function parameters (end-diastolic volume (EDV), end-systolic volume (ESV), stroke volume (SV) left ventricular ejection fraction (EF%)) [11], as already demonstrated in several cardiac stress models, e.g., in myocardial infarction model and transaortic constriction model for induction of heart hypertrophy [12, 13].

Researchers conduct in vivo studies either in one sex or less often distinguish between sexes in laboratory animal studies. Besides using various experiment ages for mice, there is still a lack of knowledge, for the most suitable time frame to perform cardiac injury models and avoid growth-dependent biases.

Therefore, this study aimed to evaluate metabolic and cardiac function parameters using serial micro PET measurements in ageing male and female mice to provide researcher data about cardiac gender differences at different age stages.

## Materials and methods

### Animals

Male and female C57/BL6 mice were purchased from Charles River (Sulzfeld, Germany). Animal care and all experimental procedures were performed according to the Guideline for the Care and Use of Laboratory Animals published by the U.S. National Institutes of Health (NIH publication no. 85–23, revised 1996). All animals received humane care. Study protocols complied with the institution's guidelines and were approved by the Government's animal ethics committee.

### In vivo cardiac PET imaging

ECG-gated 18F-FDG micro PET scans were performed on 10, 14 and 18 weeks of age, using a dedicated small-animal PET scanner (Inveon Dedicated PET, Preclinical Solutions, Siemens Healthcare Molecular Imaging, Knoxville, TN, USA; n = 8 for the male group, n = 8 for the female group) as described previously [13, 14]. The animals had free access to food and water until just before the scan, as published previously [10, 11, 14, 15]. Anaesthesia was induced (2.0–2.5%) and maintained (1.5%) with isoflurane delivered in pure oxygen at a rate of 1.4 L/min via a face mask. The mice did not receive any special pretreatment to enhance the myocardial uptake since it is well known that isoflurane anaesthesia leads to a significant myocardial 18F-FDG uptake [16–19]. The core body temperature was maintained within the normal range using a heating pad and monitored by a rectal thermometer. Neonatal ECG electrodes (3 M, St. Paul, MN, USA) were placed on both forepaws and the left hind paw. After placing an intravenous catheter into a tail vein, approx. 20 MBq of 18F-FDG was injected in a volume of ~ 100 mL. The catheter was then flushed with 50 mL of saline solution. Animals remained anaesthetized during the entire scan and were placed in prone position within the PET tomograph. A three-dimensional PET recording was obtained in list mode lasting from 30–45 min after injection of the tracer. For attenuation and scatter correction, a 7-min transmission scan was performed with a rotating [57Co] source immediately after each PET scan, as described previously [12]. Recovery from anaesthesia and the PET scan was monitored closely in the home cage with a veterinarian monitoring. The recorded data were processed with the Inveon Acquisition Workplace (Siemens Medical Solutions, Knoxville, TN, USA). 18F-FDG list-mode acquisitions were reconstructed, as described previously [12].

### PET image analysis

PET images were analyzed using the Inveon Research Workplace (Siemens Medical Solutions) as described previously [10, 20].

Inveon Research Workplace was used for assessing the percentage of the injected activity per gram (%IA/g) in the myocardium and left ventricular metabolic volume (LVMV) for the measurement of myocardial mass from static images. A cubic volume of interest (VOI) was drawn around the left ventricle, and a threshold value excluding the 30% least hottest voxels was applied. Correct VOI placement was always verified in three projections (axial, sagittal, and coronal) [13]. ECG trigger signal accuracy was retrospectively verified using in-house software programmed in MATLAB (The Mathworks, Natick, USA) [20], and heart rate during the scan was extracted.

Estimates for myocardial viability were calculated from static images as a percentage of the left ventricular surface area and automated volume measurements with QPS® (Cedars-Sinai, Los Angeles, CA, USA) using a normative database, as described previously [11, 22]. Left ventricular function parameters: EDV, ESV, the SV, and the EF, were calculated from ECG-gated images using QGS® (Cedars-Sinai, Los Angeles, CA, USA), as described previously [10, 12].

### Statistical analysis

All results were expressed as means with standard deviation. One-way and two-way ANOVA analysis with Tukey's multiple comparisons, paired and unpaired Student's t-tests were used where appropriate. For groups without normal distribution, the Wilcoxon signed-rank or the Mann–Whitney U test was applied. The differences were considered statistically significant at a P-value of 0.05.

## Results

### Increasing cardiac metabolic and ventricle volume in male mice in early to mid-adulthood

**Fig. 1:**
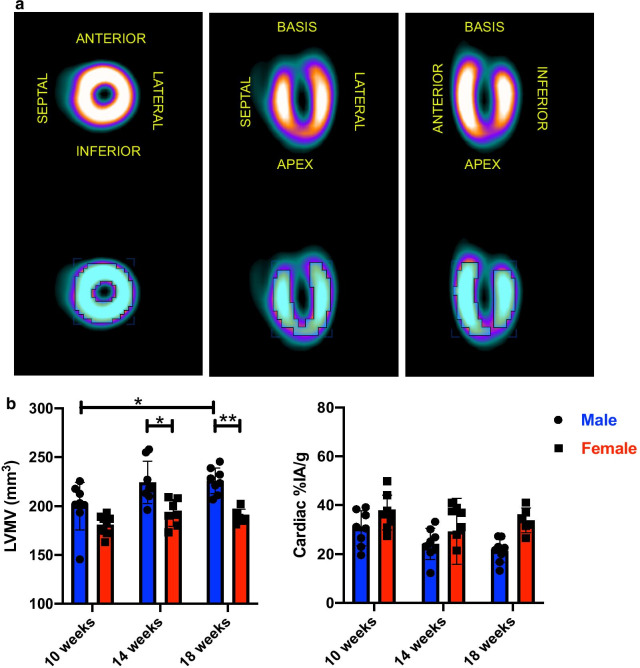
Changes in LVMV and cardiac %IA/g in ageing mice. **a** Representative cardiac PET scan images for LVMV and %IA/g are illustrated. The upper panel shows the accumulation of 18F-FDG in the left ventricle in the coronal, the horizontal long axis and the vertical long axis. The lower panel of the left ventricle shows the VOI where the 30% least hottest voxels were excluded. **b** Shows the LVMV and cardiac %IA/g for male (in blue) and female mice (in red) at 10, 14 and 18 weeks of age. All groups n = 8. All data represent mean ± SD. * p = 0.05, ** p < 0.01, *** p < 0.001

Serial micro PET scans in male mice at different time points ranging from 10 weeks, 14 weeks, and 18 weeks of age were performed to evaluate age-dependent changes in the hearts. The LVMV, which serves as a surrogate marker of the heart muscle mass (Fig. 1a upper panel), slightly increased within the first four weeks without reaching statistical significance. After this initial trend from 10 to 14 weeks, the increase in LVMV reached the level of significance in male mice at 18 weeks (10 weeks old male vs 18 weeks old male, p = 0.025, Fig. 1b left, and Table 1). Concurrent, the %IA/g resembling metabolic processes in the heart, slightly decrease over the eight weeks of follow-up (10 weeks old male vs 18 weeks old male, p = 0.058, Fig. 1a lower panel and 1B right).

Of note, although myocardial 18F-FDG uptake showed slight inhomogeneity in male mice at 14 weeks, there could be detected no significant difference in 18F-FDG uptake between the groups (please see discussion). (10 weeks old vs 14 weeks old, p = 0.462, 10 weeks old vs 18 weeks old, p = 0.985, 14 weeks old vs 18 weeks old, p = 0.449, Additional file 1: Figure S1A left).

**Table 1:** Multiparametric longitudinal PET measurements in male mice

Male mice	LVMV	EDV (µl)	ESV (µl)	SV (µl)	EF (%)	%IA/g	Weight (g)
10 weeks old	200.0 ± 24.4	36.4 ± 7.6	6.4 ± 3.2	30.1 ± 7.0	82.4 ± 9.2	30.5 ± 7.1	24.7 ± 0.8
14 weeks old	224.4 ± 21.7	39.3 ± 4.1	12.5 ± 3.1	26.8 ± 4.8	68.0 ± 7.9	24.2 ± 6.4	26.6 ± 0.9
18 weeks old	226.0 ± 13.2	42.6 ± 5.3	15.0 ± 5.5	27.8 ± 6.4	65.0 ± 13.5	21.5 ± 4.9	28.4 ± 1.7

LVMV left ventricular metabolic volume, EDV end-diastolic volume, ESV end-systolic volume, SV stroke volume, EF ejection fraction, %IA/g percentage of the injected activity per gram (determined within the VOI where the 30% least hottest voxels were excluded), bodyweight of the animals

**Fig. 2:**
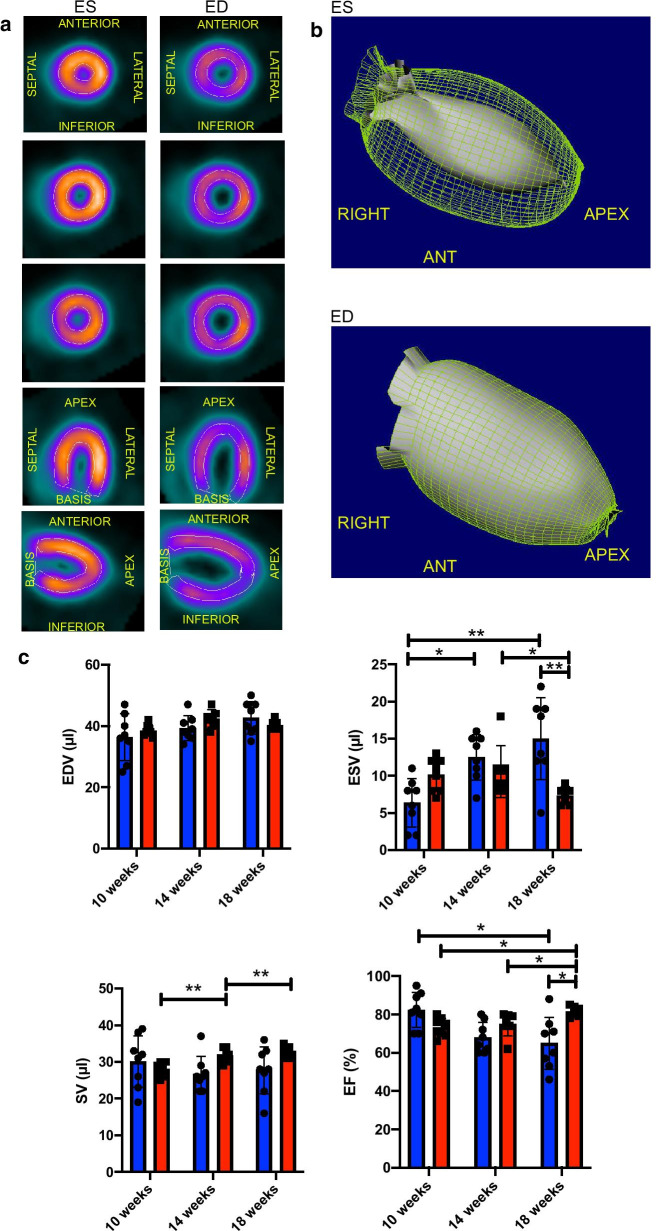
Assessment of clinically relevant cardiac function parameters by three-dimensional PET imaging. **a** shows different PET images' axes at various left ventricle levels during end-systole (left) and end-diastole (right). **b** illustrates three-dimensional reconstruction of the left ventricle in the ROA view. Upper picture: in end-systole and lower picture in end-diastole. **c** Comparison of male and female mice cardiac PET parameters at different stages of follow up. Data of male mice are illustrated in blue, and female mice are in red. All groups n = 7–8. Data represent mean ± SD. * p < 0.05, ** p < 0.01, *** p < 0.001

Functional cardiac analysis (Fig. 2a, b), showed an unchanged EDV (10 weeks old vs 18 weeks old, p = 0.242, Fig. 2c) while there was a constant increase in ESV over time (10 weeks old vs 14 weeks old, p = 0.013, 10 weeks old vs 18 weeks old, p = 0.006, Fig. 2c). While SV remained widely stable over eight weeks up (10 weeks old vs 14 weeks old: p = 0.648, 10 weeks old vs 18 weeks old: p = 0.788, 14 weeks old vs 18 weeks old: p = 0.923, Fig. 2c), there was a significant decrease in the EF% at eight weeks of follow up (10 weeks old vs 18 weeks old, p = 0.023, Fig. 2c). Already at 14 weeks of age, a strong tendency towards a reduced EF% could be detected (10 weeks old vs 14 weeks old, p = 0.052).

Summarizing these measurements, there was an age-dependent increase in LVMV and parallel increasing ESV with stable SV in male mice, indicating that male heart displays ongoing cardiac growth after ten weeks of age.

### Stable cardiac metabolic and volume equilibrium in female mice in early to mid-adulthood

In the eight weeks of serial cardiac assessment, the female mice did not show any change in the LVMV (10 weeks old vs 14 weeks old: p = 0.285, 10 weeks old vs 18 weeks old: p = 0.105, 14 weeks old vs 18 weeks old: p = 0.838, Fig. 2c and Table 2). The %IA/g uptake was constant over time (10 weeks old vs 14 weeks old: p = 0.636, 10 weeks old vs 18 weeks old: p = 0.631, 14 weeks old vs 18 weeks old: p = 0.993, Fig. 2c).

**Table 2:** Multiparametric longitudinal PET measurements in female mice

Female mice	LVMV	EDV (µl)	ESV (µl)	SV (µl)	EF (%)	%IA/g	Weight (g)
10 weeks old	181.1 ± 10.2	38.5 ± 1.9	10.1 ± 2.2	28.1 ± 2.0	73.3 ± 4.8	37.0 ± 7.2	20.7 ± 0.5
14 weeks old	193.0 ± 13.6	42.4 ± 2.9	10.6 ± 3.5	31.7 ± 1.9	75.1 ± 6.3	33.5 ± 7.0	24.0 ± 0.7
18 weeks old	189.3 ± 7.3	40.3 ± 1.6	7.3 ± 1.2	32.7 ± 1.6	81.5 ± 2.9	33.8 ± 5.0	25.4 ± 0.7

LVMV left ventricular metabolic volume, EDV end-diastolic volume, ESV end-systolic volume, SV sstroke volume, EF ejection fraction, %IA/g percentage of the injected activity per gram (determined within the VOI where the30% least hottest voxels were excluded), bodyweight of the animals

Of note, the cardiac viability was not decreased or limited at any time point (10 weeks old vs 14 weeks old: p = 0.236, 10 weeks old vs 18 weeks old: p = 0.341, 14 weeks old vs 18 weeks old: p > 0.999, Additional file 1: Figure S1 A right). Analysis of clinically relevant cardiac function parameters did not show any statistically significant changes in EDV (10 weeks old vs 14 weeks old: p = 0.079, 10 weeks old vs 18 weeks old: p = 0.344, 14 weeks old vs 18 weeks old: p = 0.073, Fig. 2c).

The ESV did not change after the first four weeks, but after eight weeks it was significantly decreased (10 weeks old vs 14 weeks old: p = 0.911, 10 weeks old vs 18 weeks old: p = 0.081, 14 weeks old vs 18 weeks old: p = 0.003, Fig. 2c).

Regarding the SV, there was an initial increase within the first four weeks of follow up, that was stable at eight weeks (10 weeks old vs 14 weeks old: p = 0.009, 10 weeks old vs 18 weeks old: p = 0.004, 14 weeks old vs 18 weeks old: p = 0.404, Fig. 2c). As a consequence of the rise in SV, the EF slightly increased over the time course of serial measurements (10 weeks old vs 14 weeks old: p = 0.588, 10 weeks old vs 18 weeks old: p = 0.014, 14 weeks old vs 18 weeks old: p = 0.032, Fig. 2c). Surprisingly, in contrast to male mice, there were no significant cardiac physiology changes in female mice.

### Gender-specific alteration in cardiac function parameters over eight weeks of follow up

As compared to female mice, LVMV was significantly higher in male mice at 14 and 18 weeks of age (14 weeks old: male vs female, p = 0.012, 18 weeks old: male vs female, p = 0.003 Fig. 1b left).

Although there was a tendency in male mice towards a lower %IA/g over time, no significant difference between the sexes at the three-time points could be observed. (10 weeks old: male vs female, p = 0.839, 14 weeks old: male vs female, p = 0.969, 18 weeks old: male vs female, p = 0.098 Fig. 1b right).

While EDV over time displayed no gender-specific differences (10 weeks old: male vs female, p = 0.935, 14 weeks old: male vs female, p = 0.754, 18 weeks old: male vs female, p = 0.935, Fig. 2c), there was a significant higher ESV in male compared to female mice at 18 weeks of age (18 weeks old: male vs female, p = 0.003, Fig. 2c), but no significant differences regarding SV at the different time points (10 weeks old: male vs female, p > 0.999, 14 weeks old: male vs female, p = 0.513, 18 weeks old: male vs female, p = 0.596, Fig. 2c). Nonetheless, a significant difference in EF% at 18 weeks of age between the genders could be observed. In contrast to their female counterparts, male mice showed a mean decreased EF by approximately 16% (18 weeks old: male vs female, p = 0.012, Fig. 2c). These findings strongly indicate that the male heart shows distinct cardiac concentric hypertrophy demonstrated by increasing LVMV, increasing ESV, and decreasing EF%. In contrast, female mice's hearts have already reached a stable steady-state at ten weeks of age.

### Bodyweight comparison in both sexes during follow up

Before each single PET scan, the mice's body weight was determined. As a result of this the body weight at the serial scans in male (10 weeks old vs 14 weeks old, p = 0.012, 10 weeks old vs 18 weeks old, p < 0.001, 14 weeks old vs 18 weeks old, p = 0.022, Additional file 1: Figure S1 B left) and female mice (10 weeks old vs 14 weeks old, p < 0.001, 10 weeks old: vs 18 weeks old, p < 0.001, 14 weeks old vs 18 weeks old, p = 0.002, Additional file 1: Figure S1 B middle) steadily increased over time. In gender comparisons, the starting body weight in male mice was significantly higher than in the age-matched female counterparts (10 weeks old: male vs female, p < 0.001), and the difference remained constant over the time course of the experiments (18 weeks old: male vs female p < 0.001, Additional file 1: Figure S1 A right). Interestingly, we could also detect a positive correlation of the mice body weight of all animals (male and female mice at 10, 14 and 18 weeks of age) to LVMV (r = 0.6441, p < 0.001, Additional file 1: Figure S1 C).

### ECG tracking of both genders showing reduced heart rates in male mice, correlations to the percentage injected activity in the heart and left ventricular metabolic volume

**Fig. 3:**
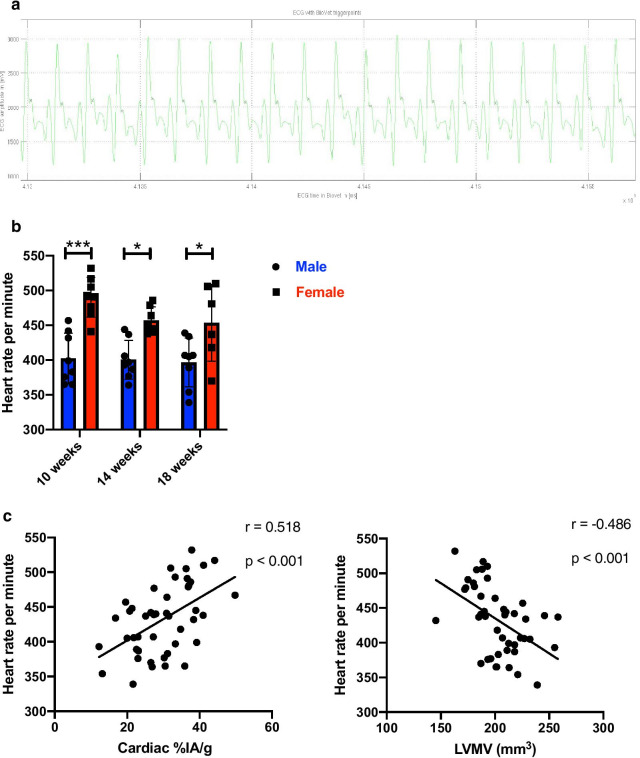
ECG analysis in both genders. **a** depicts the ECG tracking during the PET scan. Small black crosses in the QRS complex demonstrate the time of the trigger event. **b** ECG assessment of the heart rate in both genders during the PET scans. **c** Correlation of heart rate with cardiac LVMV and %IA/g, respectively. All groups n = 7–8 at different time points and cumulative group size in correlations n = 45. All data represent mean ± SD. * p < 0.05, ** p < 0.01, *** p < 0.001.

During the gated cardiac PET scan (further explanation in the methodical section), ECG data were recorded (Fig. 3a). As a result of this female mice showed a stable and significantly higher heart rate per minute than their male counterparts (10 weeks old: female: 490.4 ± 28.9 vs male: 402.4 ± 36.5, p < 0.001, Fig. 3b). Furthermore, this difference was statistically stable after the 8th week of the follow up (18 weeks old: female: 453.7 ± 55.2 vs male: 396.8 ± 35.2, p = 0.044, Fig. 3b).

Furthermore, heart rate showed strong, significant positive correlation with %IA/g (r = 0.518, p < 0.001, Fig. 3c left). In concordance to this observation, the LVMV displays a moderate but significant negative correlation the heart rate (r = −0.486, p < 0.001, Fig. 3c right). Of note, we could detect a statistically significant negative correlation of the documented heart rate to the body weight (r = −0.549, p < 0.001, Additional file 1: Figure S1 D).

## Discussion

In this study, we provide evidence for age-dependent gender-specific differences in murine hearts by small-animal PET imaging.

By primarily assessing the left ventricular metabolic volume, we used a standardized method that provides an efficient and proven surrogate marker of murine heart mass [13]. Our results indicate that male hearts compared to female hearts are bigger in LVMV and that growth of male hearts continues after ten weeks of age. Here we show that the female heart increases only mildly and not statistically significant in the LVMV. Our results mirror human data showing that males' heart mass is up to 15–30% higher [23]. It is known as well that male and female hearts show hypertrophy during ageing [24]. Yet our study did only document the murine early to mid-adulthood.

Regarding the clinically relevant cardiac parameters, the ESV in male increases over time. The SV remains stable, which is in line with the observation of growing male hearts [24]. While the male hearts could become more durable, they are in theory, not dependent on the former ventricle volume to maintain the body perfusion. Therefore, as a consequence, the ejection fraction in male mice would drop. While the female hearts do not increase in LVMV at these stages of age, body mass increases over time. They could depend on more ESV reserve, and therefore, the EF% could increase for adequate organ perfusion.

The %IA/g, also described as %ID/g in previous publications resembles the ratio between the activity of the tracer detected in the tissue, and the total tracer activity injected [14, 19, 25]. Interestingly, the %IA/g in male mice tends to decrease over follow-up time. In contrast, the cardiac %IA/g remains stable in the female mice, which could be partially attributed to the moderate increase in body weight and the constant cardiac mass. Interestingly, our data indicate a positive correlation of %IA/g and the measured heart rate, which could illustrate a higher cardiac demand for glucose at higher heart rates. However, the %IA/g could depend on multiple distinct and yet undetermined variables masking a direct affiliation.

Of note, the term %ID/g or more precisely %IA/g was primarily described in post-myocardial injury studies.

These recent studies could demonstrate that the cardiac %IA/g in myocardial infarction is elevated in the acute phase on day five in humans [25]. This increased accumulation could be associated with the inflammatory response, and the invading immune cells and subsequent higher glucose consumption in the tissue [11, 14]. Herein, we could demonstrate that the uptake of 18F-FDG also positively correlates with murine heart rate. Our results also support the notion that increasing heart mass in healthy hearts, represented by LVMV, correlates with reduced heart rates. The documented murine heart rate and correlation towards the cardiac surrogate marker LVMV, even with isoflurane narcotic, is in line with published studies [26][27]. Of note, we cannot exclude sex differences in response to anaesthesia, since the standard mouse model is male and more research is warranted to provide insight.

A limitation of the 18F-FDG PET scan could be the usage of isoflurane, as discussed in [16]. Concerning the usage of 18F-FDG, cardiac uptake and metabolism could be modified by various systemic factors, e.g., insulin and glucagon that were not evaluated in this study [17].

This study provides insight into the cardiac homeostasis at 10 weeks, 14 weeks, and 18 weeks of age in male and female mice using the innovative design of serial non-invasive PET imaging. Our results could help research groups determine the correct age choice for their murine injury model in both sexes and avoid growth-related biases.

## Supplementary information

**Additional file 1.**. **Figure S1**: (**a**) Viability defect in male and female mice at different weeks of age. (**b**) is showing the change in weight during the follow-up. (**c**) Correlation of mice body weight and LVMV. (**d**) Correlation of heart rate per minute and body weight in male and female mice. All groups n = 7-8 at different time points and cumulative group size in correlations n = 45. All data represent mean ± SD. * p < 0.05, ** p < 0.01, *** p < 0.001.

## Data Availability

The authors confirm that the data supporting the findings of this study are available within the article and/or its supplementary materials.
