# Supplementary figures and images for: Comparison of metabolic and functional parameters using cardiac 18F-FDG-PET in early to mid-adulthood male and female mice
Source: EJNMMI Res. 2021 Jan 19;11:7. doi: 10.1186/s13550-021-00748-z (PMC7815863; doi:10.1186/s13550-021-00748-z)

**A**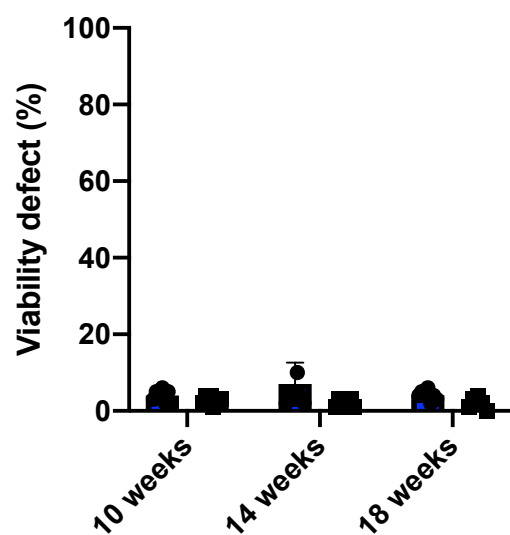**B**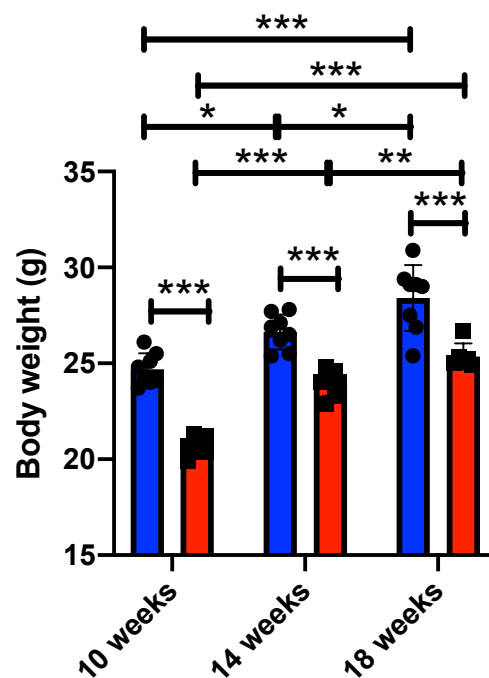**C**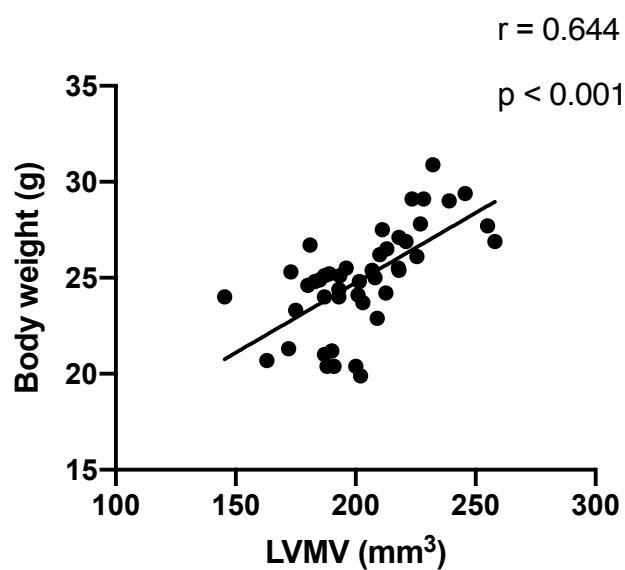**D**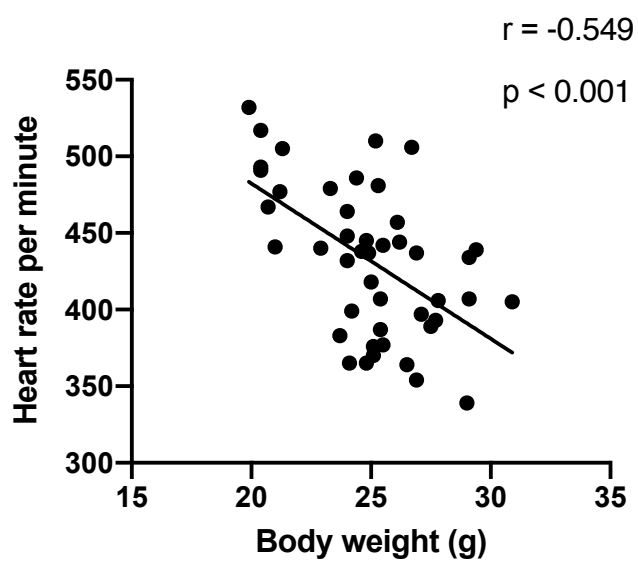

Supplement: Supplementary file 1 — Additional file 1.. Figure S1: (a) Viability defect in male and female mice at different weeks of age. (b) is showing the change in weight during the follow-up. (c) Correlation of mice body weight and LVMV. (d) Correlation of heart rate per minute and body weight in male and female mice. All groups n = 7-8 at different time points and cumulative group size in correlations n = 45. All data represent mean ± SD. * p < 0.05, ** p < 0.01, *** p < 0.001. [file 13550_2021_748_MOESM1_ESM.pdf]
